# Facial cellulitis revealing choreo-acanthocytosis: a case report

**DOI:** 10.11604/pamj.2014.17.322.4085

**Published:** 2014-04-28

**Authors:** Younes Samia, Cherif Yosra, Bellazreg Foued, Aissi Mouna, Berriche Olfa, Souissi Jihed, Braham Hammadi, Frih-Ayed Mahbouba, Letaief Amel, Sfar Mohamed Habib

**Affiliations:** 1 Department of Endocrinology and Internal Medicine, Laboratory of Hematology, Tahar Sfar University Hospital of Mahdia, Mahdia, Tunisia; 2 Department of Infectious Diseases, Farhat Hached University Hospital of Sousse, Sousse, Tunisia; 3 Department of Neurology, Fattouma Bourguiba's University Hospital of Monastir, Monastir, Tunisia

**Keywords:** Choreo-acanthocytosis, facial cellulitis, dyskinetic movements

## Abstract

We report a 62 year-old-man with facial cellulitis revealing choreo-acanthocytosis (ChAc). He showed chorea that started 20 years ago. The orofacial dyskinisia with tongue and cheek biting resulted in facial cellulitis. The peripheral blood smear revealed acanthocytosis of 25%. The overall of chorea, orofacial dyskinetic disorder, peripheral neuropathy, disturbed behavior, acanthocytosis and the atrophy of caudate nuclei was suggestive of a diagnosis of ChAc. To our knowledge no similar cases of facial cellulitis revealing choreo-acanthocytosis (ChAc) were found in a review of the literature.

## Introduction

Neuroacanthocytosis (NA) is a group of uncommon heterogenous neurodegenerative processes associated with red cell acanthocytes in the peripheral blood smear [1, 2]. Its spectrum includes various conditions which have many similarities: chorea-acanthocytosis (ChAc), McLeod syndrome, Huntington disease-like, abetalipoproteinemia, and pantothenate kinase-associated neurodegeneration [2–5]. Chorea-acanthocytosis is an autosomal recessive disorder characterized by marked orofacial dyskinesia, peripheral neuropathy, seizures, changed behavior and acanthocytes with a normal level of lipoproteins [1–3]. It is well recognized that ChAc may induce dyskinetic movements involving the oral group of muscles and can result in significant injury of lips and tongue. However, it is uncommon that the occurrence of repeated injury tongue and lips may result in severe facial cellulitis. In this report, we present a case of an adult male with severe dyskinetic disorder who had recurrent laceration to tongue and cheeks and developed a severe facial cellulitis. To our knowledge no similar cases were found in a review of the literature.

## Patient and observation

**Figure 1:**
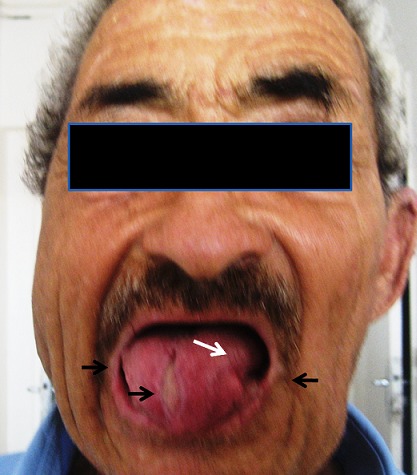
The patient photo one month after cellulite treatment, showed tongue's dystonia (white arrow) and sequel of mutilation of the tongue and lips (black arrows)

**Figure 2:**
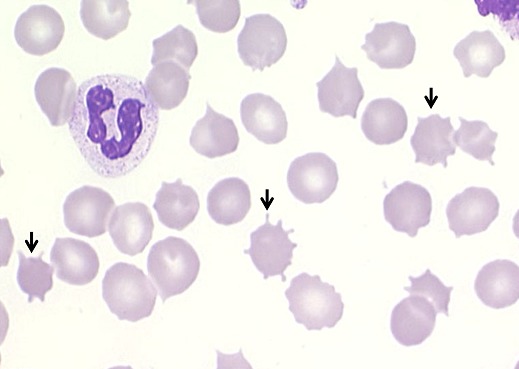
Acanthocytes (black arrows) in the peripheral blood smear of the patient

A 62 year-old-man, born to consanguineous healthy parents, was admitted on June 2013 to the department of Internal Medicine, with complaints of fever, weakness which had begun abruptly a few days ago. The blood pressure was 120/70 mm of mercury, the pulse rate 86 and respirations 16 per minute, the temperature at 39°C. Physical examination also revealed unilateral flat, well-demarcated, indurated and red rash on the right cheek. He showed limb chorea and that spread gradually throughout the entire body associated with orofacial dystonic movements especially in the tongue, causing dysarthria and serious dysphagia with resultant weight loss. Oral dystonia resulted in recurrent mutilation of the tongue and lips (Figure 1). He had multiple lacerations, bites and scars on the lateral edges of the tongue and inside the cheeks. A thorough neurological examination showed hyporeflexia, hypotonia, distal muscle atrophy and edema in both lower limbs. The remainder of her physical examination including the pharynx, tympanic membranes, cervical nodes, heart, lungs, and abdomen is normal. According to the patient′s sisters, he was the only affected sibling, his birth and childhood development was uneventful and the movement disorder had started 20 years ago. He had progressive dysphagia, difficulty in chewing and swallowing food. These movements led to petulance and social avoidance. The blood smear showed the presence of more than 25% acanthocytes (Figure 2). Noteworthy laboratory findings revealed normal CPK and LDH level, the absence of Kell antigens in the red blood cell membrane.

**Figure 3:**
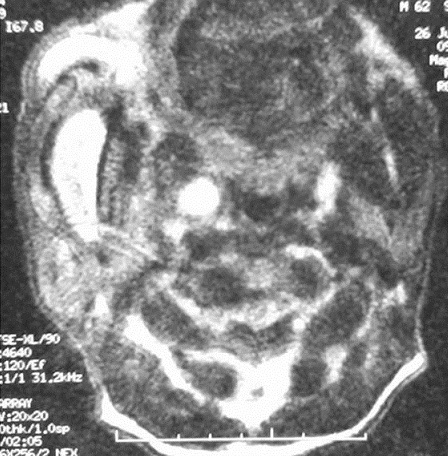
Cervical MRI axial FAT SAT T2 section showed abscess 40 x 20 mm of the right parotid gland (black arrow)

Blood tests showed moderately elevated erythrocyte sedimentation rate, elevated C reactive protein level, an increased white blood cell count. Two specimen of blood culture were positive for methicillin-sensitive *Staphylococcus aureus*. The cervical MRI provided imaging of a 4x2 cm abscess of the right parotid gland, with of lesions in facial tissues (Figure 3). Cerebral CT scan revealed an atrophy of caudate nuclei. The electromyography enclosed axonal sensori-motor neuropathy. Fundus examination showed no pigmentary retinopathy. The overall of chorea, orofacial dyskinetic disorder, peripheral neuropathy, disturbed behavior, RBC acanthocytosis and the atrophy of caudate nuclei was suggestive of a diagnosis of ChAc. The diagnosis of cellulitis revealing ChAc was made. He was started on antibiotics: Oxacillin: 8 g/day associated with Rifampicin: 1200 mg/day during 14 days. Then, he was treated with Sulfamethoxazole, Trimethoprime: 2400 mg/day and Rifampicin: 1200 mg/day during 8 weeks. The celllitis resolved totally. Haloperidol was prescribed with a moderate improvement in clinical status, the movement disorder and injuries to the tongue mildly decreased.

## Discussion

We report a case of ChAc associated with facial cellulitis. Although ChAc has been associated with many disorders [2], it has not been described in association with facial cellulitis. Choreo-acanthocytosis is estimated that over 1000 cases exist all over the world [3]. It has been reported in child and young adult [1, 2, 4, 6–8] unlike the present case. Our patient had typical ChAc with chorea, tongue′s dystonia, dysphagia, cognitive impairment and peripheral axonal neuropathy [3, 7, 8]. Conversely, he did not present clinical features of heart disease, Kell antigen, characteristics of Mac Leod syndrome [3, 5, 7].

CT scan and MRI showed usually atrophy of the striata, caudate nucleus, and increased signal intensity on T2-weighted imaging [3, 6, 9, 10]. Brain perfusion SPECT may reveal a decrease in blood flow in the basal ganglia prior to the atrophy of the caudate nuclei [6, 11]. CT scan demonstrated atrophy of the caudate nucleus in our patient.

Although ChAc has been associated with many disorders [2, 12], it has not been described in association with facial cellulitis. Facial cellulitis presents an infection of the soft tissues of the face. It, nonetheless, requires accurate diagnosis and prompt treatment, as the infection can lead to serious complications [13–15]. The most common predisposing factors are the infection of the paranasal sinuses, orbital structures, upper respiratory infection and recent trauma or surgery [13, 14]. In our case, it results from the extensive laceration due to the orofacial dystonia.

The diagnosis and the management of ChAc remain yet tricky. Medical treatment including botulinum toxin an injections and atypical neuroleptic drugs may improve the movement disorder [2]. Deep brain stimulation of the striata has been tried with variable results [2,16]. Our patient experienced a moderate improvement with neuroleptic drugs. Although it was not possible to perform specific investigations in this patient, the history of massive edema, lowered plasma albumin level and the absence of renal and liver disease support that our patient experienced a protein-losing enteropathy in association with facial cellulitis and ChAc. This case report highlights first, the significance of peripheral blood smear check in all cases of severe dyskinetic disorders and the paramount treatment to reduce infectious outcomes.

## Conclusion

In summary, we have reported here in the case of facial cellulitis revealing ChAc. Cervico-facial cellulitis is not an uncommon condition with the potential and life-threatening complications. An understanding of predisposing factors, microbiology is essential to its management. Without treatment, ChAc may be disabling and may contribute to this infection.

## References

[1] Rampoldi L, Danek A, Monaca AP (2002). Clinical features and molecular bases of neuroacanthocytosis. J Mol Med (Berl).

[2] Jung HH, Danek A, Walker RH (2011). Neuroacanthocytosis syndromes. Orphanet J Rare Dis..

[3] Gövert F, Schneider SA (2013). Huntington's disease and Huntington's disease-like syndromes: an overview. Curr Opin Neurol..

[4] Bayreuther C, Borg M, Ferrero-Vacher C, Chaussenot A, Lebrun C (2010). Chorea-acanthocytosis without acanthocytes. Rev Neurol (Paris).

[5] Danek A, Jung HH, Melone MA, Rampoldi L, Broccoli V, Walker RH (2005). Neuroacanthocytosis: new developments in a neglected group of dementing disorders. J Neurol Sci..

[6] Boughammoura-Bouatay A, Karmani M, Chebel S, Frih-Ayed M (2005). Neuroacanthocytosis and epilepsy. Rev Med Interne..

[7] Danek A, Walker RH (2005). Neuroacanthocytosis. Curr Opin Neurol..

[8] Hardie RJ, Pullon HW, Harding AE (1991). Neuroacanthocytosis: A clinical, haematological and pathological study of 19 cases. Brain..

[9] Nicholl DJ, Sutton I, Dotti MT, Supple SG, Danek A, Lawden M (2004). White matter abnormalities on MRI in neuroacanthocytosis. J Neurol Neurosurg Psychiatry..

[10] Katsube T, Shimono T, Ashikaga R, Hosono M, Kitagaki H, Murakami T (2009). Demonstration of Cerebellar Atrophy in Neuroacanthocytosis of 2 Siblings. AJNR Am J Neuroradiol..

[11] Ichiba M, Nakamura M, Kusumoto A (2007). Clinical and molecular genetic assessment of a chorea-acanthocytosis pedigree. J Neurol Sci..

[12] Walterfang M, Evans A, Looi JC (2011). The neuropsychiatry of neuroacanthocytosis syndromes. Neurosci Biobehav Rev..

[13] La Rosa J, Bouvier S, Langeron O (2008). Prise en charge des cellulites maxillo-faciales. Le Praticien en Anesthésie Réanimation..

[14] Chaudhry IA, Shamsi FA (2007). Outcome of treated orbital cellulitis in a tertiary eye care center in the middle East. Ophthalmology..

[15] Kouassi YM, Janvier B, Dufour X, Bouche G, Klossek JM (2011). Microbiology of facial cellulitis related to dental infection. Med Mal Infect..

